# Glucose-Regulated Protein 78 Signaling Regulates Hypoxia-Induced Epithelial–Mesenchymal Transition in A549 Cells

**DOI:** 10.3389/fonc.2019.00137

**Published:** 2019-03-12

**Authors:** Ling-Ling Sun, Chang-Ming Chen, Jue Zhang, Jing Wang, Cai-Zhi Yang, Li-Zhu Lin

**Affiliations:** Integrative Cancer Centre, the First Affiliated Hospital of Guangzhou University of Chinese Medicine, Guangzhou, China

**Keywords:** lung cancer, lung adenocarcinoma, epithelial mesenchymal transition, hypoxia, glucose-regulated protein 78, GRP78

## Abstract

**Objective:** Metastasis and therapeutic resistance are the major determinants of lung cancer progression and high mortality. Epithelial–mesenchymal transition (EMT) plays a key role in the metastasis and therapeutic resistance. Highly expressed glucose-regulated protein 78 (GRP78) is a poor prognostic factor in lung cancer and possibly correlated with EMT. This study aims to examine whether the up-regulation of GRP78 is involved in EMT in lung adenocarcinoma and explore the underlying downstream molecular pathways.

**Study Design:** EMT was assessed by analysis of cell morphology and expression of EMT protein markers in A549 cells under normoxia, hypoxia and silencing GRP78 conditions. The expression levels of Smad2/3, Src, and MAPK (p38, ERK, and JNK) proteins were examined by Western blot analysis under hypoxia and treatments with phosphorylation inhibitors.

**Results:** Under hypoxic conditions, the EMT morphology significantly changed and the GRP78 expression was significantly up-regulated in A549 cells compared with those in normoxia control. The expression and phosphorylation levels of smad2/3, Src, p38, ERK, and JNK were also upregulated. When GRP78 was silenced, EMT was inhibited, and the levels of phospho-smad2/3, phospho-Src, phospho-p38, phospho-ERK, and phospho-JNK were suppressed. When the activation of Smad2/3, Src, p38, ERK, and JNK was inhibited, EMT was also inhibited. The inhibition effect on EMT by these phosphorylation inhibitors was found to be weaker than that of GRP78 knockdown.

**Conclusions:** Hypoxia-induced EMT in A549 cells is regulated by GRP78 signaling pathways. GRP78 promotes EMT by activating Smad2/3 and Src/MAPK pathways. Hence, GRP78 might be a potential target for treatment of lung adenocarcinoma.

## Introduction

Lung cancer is the leading cause of cancer death worldwide; according to the estimated data from GLOBOCAN in 2012, one of five cancer deaths is due to lung cancer (1.59 million deaths, 19.4% of the total cancer deaths) (1). Despite significant progress in the development of new therapies for lung cancer, metastasis and therapeutic resistance remain the major determinants of lung cancer progression and high mortality (2).

Mounting evidence demonstrated that epithelial–mesenchymal transition (EMT) is involved in the metastasis and therapeutic resistance of lung cancer. EMT refers to the biological process by which epithelial cells are transformed into mesenchymal phenotypes through specific procedures. EMT inhibits the expression of E-cadherin and cytokeratin in epithelial cells, upregulates the expression of N-cadherin and vimentin in mesenchymal cells and promotes the ability of cells to secrete matrix metalloproteinase and fibronectin. EMT can be induced by various factors, such as TGF-beta, which increases the expression of key nuclear transcription factors including Twist, Snail and ZEB (3) and causes phenotypic changes by activating intrinsic cellular signal molecules including Src, MAPK, Smad2/3, and other signals (4–6).

Hypoxia is a common hallmark of several human malignancies and an independent and unfavorable prognostic factor associated with the occurrence of EMT (7–9). Previous studies found that cAMP-dependent protein kinase, hypoxia factor Hif-1alpha(HIF1a) and unfolded protein response can potentiate EMT; moreover, treatment with insulin-like growth factor 1 receptor inhibitor reverses hypoxia-induced EMT (8–11), However, the mechanisms of hypoxia-induced EMT remain unknown. Understanding the biology of hypoxia-induced EMT and their implications in therapeutic relapse may provide new crucial approaches for development of improved therapeutic strategies.

The 78-kDa glucose-regulated protein (GRP78), also known as BiP and HSPA5, is highly expressed in many types of cancers, including lung, hepatocellular cancer, and breast cancer (12–15). It could inhibit apoptosis of cancer cells, and induce chemoresistance of cancer (16–18). What's more, it is closely related to EMT. Zhang et al. (19) reported that high expressed GRP78 induced EMT in hepatocellular carcinoma cell lines. Lizardo et al. (20) that up-regulation of GRP78 in metastatic cancer cells is necessary for lung metastasis in some highly metastatic cell line models, such as osteosarcomas and murine mammary adenocarcinoma. Zhang et al. (21) demonstrated that overexpressing GRP78 facilitated the expression and secretion of TGF-beta1, which further activated EMT. However, Chang et al. (22) stated that overexpressing GRP78 inhibited the metastasis of colon cancer through EMT biomarkers. Thus far, the relationship between GRP78 expression and EMT remains controversial. Whether GRP78 expression has causality link with EMT in lung cancer also remains unknown. Hence, the present study aims to examine the role of up-regulation of GRP78 in EMT in lung adenocarcinoma and explore the downstream molecular pathways involved.

## Methods

### Cell Culture and Conditioning

Human lung adenocarcinoma A549 cells were purchased from the Cell Bank of the Chinese Academy of Sciences (Shanghai, China) and cultured in RPMI-1640 medium (Gibco, USA) supplied with 10% FBS (Gibco, USA) and 100 U/ml penicillin/streptomycin in 5% CO_2_ incubator at 37°C. The medium was changed every 3 days. The cells were treated with normal O_2_ as control.

A549 cells were cultured with 2% O_2_ for hypoxia condition. The concentration of protein inhibitors for treatment of A549 cells were as follows: 550 nM SB505124 (phospho-Smad2/3 inhibitor), 25 nM KX2-391 (phospho-Src inhibitor), 100 nM JNK-IN-8 (phospho-JNK inhibitor), 2.5 μM SB203580 (phospho-p38 inhibitor), and 1.5 μM FR180204 (phospho-ERK). All of the inhibitors were purchased from Celleck Chemicals (USA).

### Assessment of Cell Morphology

Morphological changes were examined using phase-contrast microscopy (Olympus, Japan).

### Real-Time Quantitative Fluorescent PCR

Experiments were performed following the methods in our previous study. The cells were collected to extract total RNA using Trizol method. The cDNA was synthesized with Prime Script TMRT Master Mix (RR036A; Takara, Japan) through reverse transcription and used as template to amplify target genes with real-time quantitative fluorescent PCR with SYBR^®^ Premix Ex TaqII (RR820A; Takara, Japan). The specific primers (Invitrogen, USA) of each transcription factor (Snail1, Snail2, Twist, ZEB1 and ZEB2) were also based on such study (23). The reaction condition was 95°C for 30 s, followed by 95°C for 5 s and 60°C for 30 s with 40 cycles. The amplified productions were quantitatively analyzed with 2-ΔΔCt method. All tests were repeated three times.

### Western Blot Analysis

Experiments were performed following the methods in our previous study. The specific program and concentration of each antibody were also based on such study (23). Briefly, the protein was extracted with PIRA buffer and centrifuged at 12,000 g for 15 min at 4°C. Fifty microgram total proteins were separated with 10%SDS-PAGE. After electrophoresis, proteins were blotted to polyvinylidene fluoride (PVDF) membranes and then blocked with 5% skim milk powder with 0.1% Tween-20. The blots were then probed at 4°C overnight with the relevant primary antibodies respectively, and incubated in 4°C for overnight. The membranes were rinsed with TBST for 3 times, 10 min each time. Then secondary goat anti-rabbit or anti-mouse IgG-HRP antibodies were added for incubation in room temperature for 2 h. The membranes were rinsed with TBST for 3 times, and 10 min per time. Then the membranes were developed with ECL (Beijing Kangwei Biotech, China) and taken photos to analyze the relative expression of proteins with GAPDH as internal referral. All tests were repeated three times.

### Immunofluorescence Staining

Cells were cultured on six-well chamber slides for immunofluorescent staining. The cells were fixed in 4% paraformaldehyde for 30 min at room temperature. After washing with PBS three times for 10 min each time, the cells were permeabilised with 0.1% Triton X-100 in PBS for 15 min. After three washes with PBS, the cells were blocked with 5% BSA for 30 min at room temperature. The cells were incubated with the indicated GRP78 antibody (1:250) overnight at 4°C, washed three times with PBS and incubated with fluorescent secondary antibodies. Nuclear staining was performed in the dark with DAPI at room temperature. Phase contrast and fluorescent microscopy was performed using an NikonTi-U Inverted Fluorescence Microscope (Nikon, Japan).

### Plasmid Transfection and Identification

GRP78 short hairpin RNA(GRP78shRNA) eukaryotic expression plasmid was designed and synthesized by Invitrogen, USA. Transfection and identification were conducted according to the protocol of Lipofectamine 3000. using previously published methods (24). Briefly, 7.5 μl Lipofectamine 3000 Reagent was diluted with 125 μl Opti-MEM media, then blended with a diluted plasmid DNA which diluted by 10 μl P3000 Reagent and 125 μl Opti-MEM media. After incubating for 5 min, the overall mixture was added into the culture cells and cultured for 72 h. The cells carrying green fluorescence are plasmids transfected successfully in inverted microscope. 20 μg/ml blasticidin was used to screen the cells and the maintenance concentration of blasticidin is 10 μg/ml. Blank shRNA was used as a control. The mRNA and protein expression levels of GRP78 decreased by 70 and 85.33%, respectively, suggesting successful transfection.

### Statistical Analysis

Data were expressed as mean ± SD. Comparison between two groups was performed with *t*-test for independent samples. Comparison among multiple groups was performed with one-way ANOVA following LSD (equal variances) or Dunnett's *t*-test (unequal variances). P < 0.05 was set as the significance level. All analyses were performed using SPSS 22.0 software.

## Results

### Activation of EMT by Hypoxia in A549 Cells

**Figure 1:**
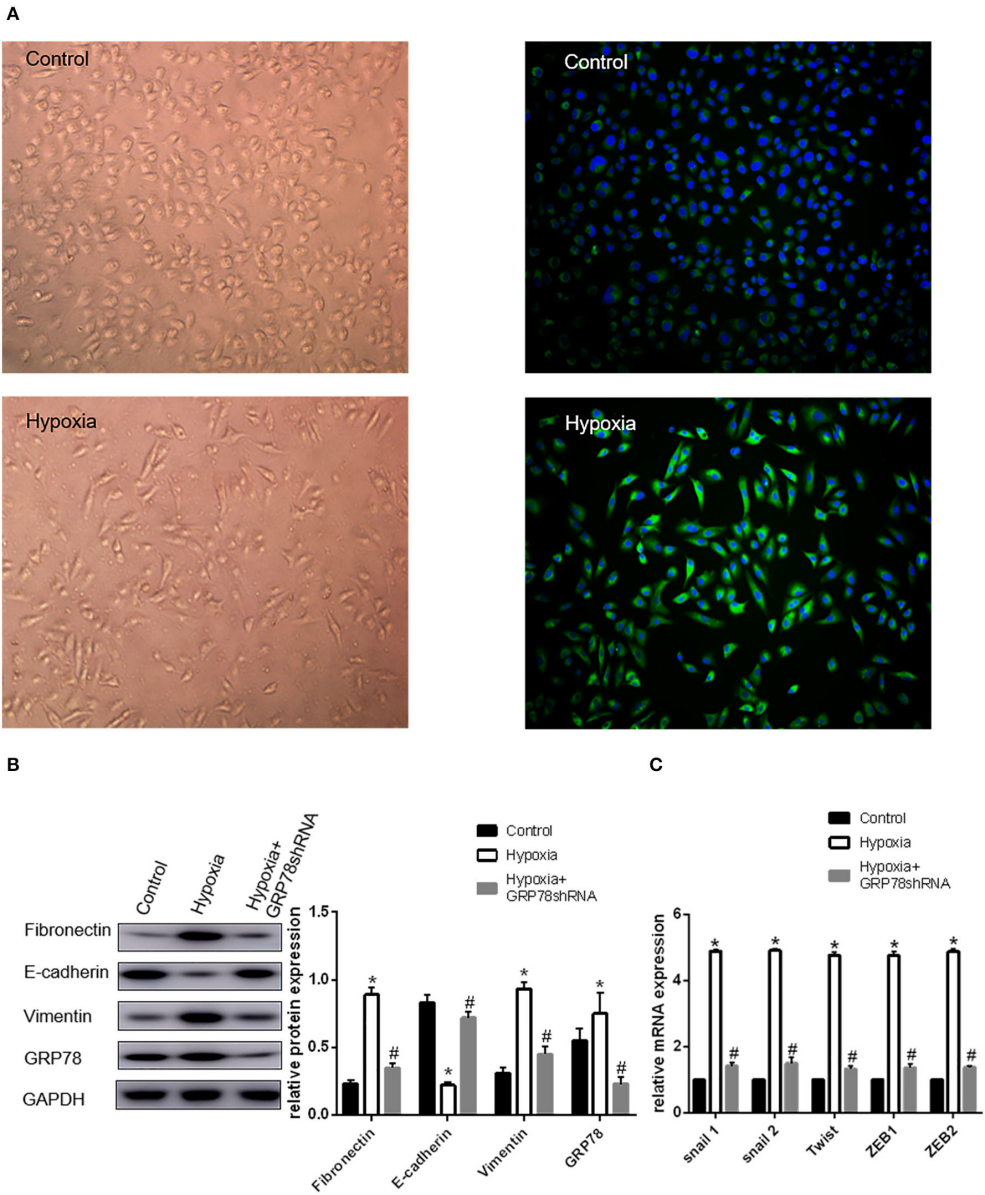
Up-regulation of GRP78 plays an important role in hypoxia-induced EMT in A549 cells. **(A)** A549 cells acquire spindle-shaped mesenchymal morphology after 72 h of 2% O_2_ hypoxia (left, 100 ×). GRP78 (green fluorescence) is highly expressed in A549 cells with spindle-shaped mesenchymal morphology (right, 100 ×). **(B)** EMT-related markers (E-cadherin, Vimentin and Fibronectin) and GRP78 were examined by Western blot analysis (left). GAPDH was used as internal control. The protein relative value (GAPDH) is plotted in the right panel (mean ± SD in three separate experiments). *P < 0.05, compared with A549 cells under the condition of normal oxygen, the expression of E-cadherin decreases, while those of Vimentin and Fibronectin increase in A549 cells under hypoxia (2% O_2_ 72 h). The expression of GRP78 also increases in A549 cells under hypoxia. #*P* < 0.05, compared with the A549 cells under the condition of hypoxia; the expression of E-cadherin increases, and those of Vimentin and Fibronectin decrease in GRP78 knockdown A549 cells under hypoxia. **(C)** EMT-related genes including Snail1, Snail2, Twist, ZEB1, and ZEB2 were examined by real-time quantitative PCR; mRNA expression relative value (control group) is plotted (mean ± SD in three separate experiments). *P < 0.05, compared with A549 cells in the control group, the mRNA expression levels of EMT-related genes including Snail1, Snail2, Twist, ZEB1, and ZEB2 increase under hypoxic condition (2% O_2_ 72 h); #*P* < 0.05, compared with A549 cells under the condition of hypoxia, the mRNA expression levels of EMT-related genes decrease in GRP78 knockdown A549 cells under hypoxia.

A549 cells cultured under hypoxia condition for 72 h showed morphological changes, from oblate fusiform-shaped epithelial cells to elongated spindle-shaped mesenchymal cells (Figure 1A). The expression levels of EMT-related genes including Snail1, Snail2, Twist, ZEB1, and ZEB2 were increased by approximately three times under hypoxic condition compared with that in the control group. The Western blot analysis showed that the protein expression of E-cadherin (biomarker of epithelial phenotype) under hypoxia found to be approximately three times less than that in the control group. The expression levels of vimentin and fibronectin (biomarker of mesenchymal phenotype) were increased by 1.48 and 1.22 times, respectively, under hypoxia condition compared with that in the control group (*P* < 0.05 compared with Normoxia, Figures 1B,C).

### Expression of GRP78 Under Normoxia and Hypoxia Conditions

The expression and location of the GRP78 protein in A549 cells under hypoxia and normoxia conditions were determined by immunofluorescence staining. Under normoxia condition, GRP78 (green fluorescence) showed weak staining intensity and was mainly distributed in the cytoplasm (Figure 1A). By contrast, under hypoxia, A549 cells showed an elongated spindle-shaped mesenchymal phenotype, and GRP78 showed strong staining intensity and was mainly distributed in the cytoplasm and cell membrane (Figure 1A). The Western blot analysis showed that the expression of GRP78 in A549 cells under hypoxia was found to be 1.36 times more than that under normoxia (Figure 1B).

### Effect of GRP78 Knockdown on the Expression of EMT Markers

The expression of GRP78 in GRP78 knockdown A549 cells under hypoxia was reduced by 70% compared with that under hypoxia. In A549 cells transfected with GRP78 shRNA under hypoxia, the expression levels of vimentin and fibronectin significantly decreased by 52 and 60%, respectively. Meanwhile, the mRNA expression levels of transcription factors (Snail1, Snail2, Twist, ZEB1, and ZEB2) were significantly inhibited under hypoxia condition and decreased by approximately 70% compared with that in the normoxia group (Figures 1B,C). The significant change in the expression of EMT biomarkers and its transcription factor mRNAs after GRP78 knockdown indicated that GRP78 might play an important role in hypoxia-induced EMT.

### Expression of Smad2/3, Src, p38, ERK and JNK in A549 Cells Under Hypoxia Condition

**Figure 2:**
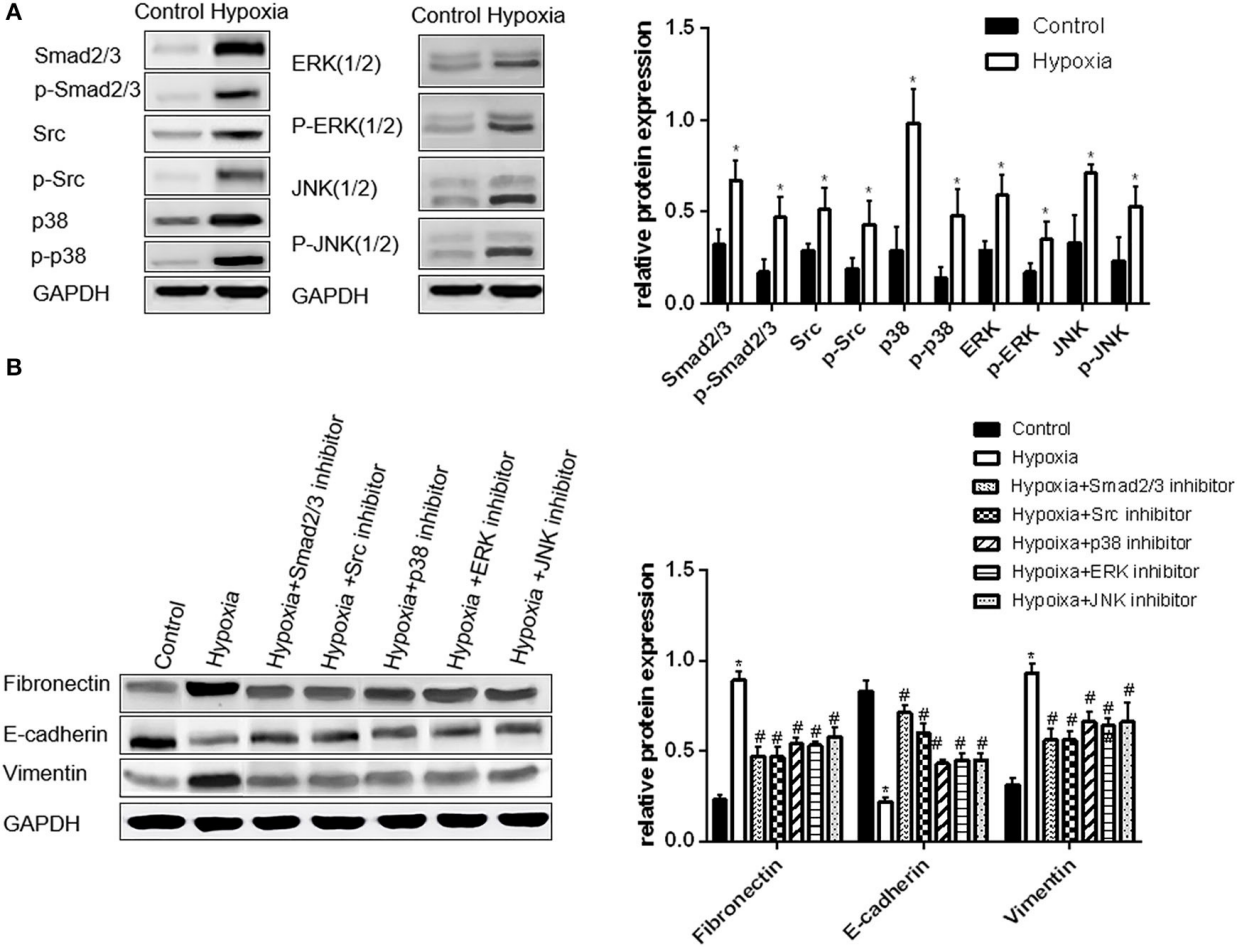
Activation of Smad2/3, Src, p38, ERK, and JNK is important in hypoxia-induced EMT in A549 cells. **(A)** Smad2/3, Src, p38, ERK, JNK, and their phosphorylated forms were examined by Western blot analysis (left). GAPDH was used as internal control. The protein relative value (GAPDH) is plotted in the right panel (mean ± SD in three separate experiments). *P < 0.05, compared with A549 cells in the normal oxygen environments, the Smad2/3, Src, and MAPK proteins of A549 cells are highly regulated and activated in hypoxia environments. **(B)** EMT markers were examined by Western blot analysis (left). GAPDH was used as internal control. The protein relative value (GAPDH) is plotted in the right panel (mean ± SD in three separate experiments). *P < 0.05, compared with A549 cells in the normal oxygen environments, the EMT process of A549 cells under hypoxia is activated; #*P* < 0.05, compared with A549 cells in the hypoxia environments, the EMT process of A549 cells under hypoxia is inhibited separately by Smad2/3, Src, p38, ERK, and JNK inhibitors. The expression levels of Fibronectin and Vimentin decrease, and that of E-cadherin increases.

The expression levels of phosphorylated Smad2/3, p38, and JNK in A549 cells significantly increased by approximately 2.4 times under hypoxia condition compared with those under normoxia condition, whereas the levels of the phosphorylated Src and ERK increased by approximately 1.8 times (all *p* < 0.05, Figure 2A). Hence, these signaling pathways were activated under hypoxia condition.

The inhibitors of Smad2/3, Src, p38, ERK, and JNK were used to treat A549 cells under hypoxia condition to further verify the relationship between these signaling molecules with hypoxia-induced EMT. The expression levels of EMT protein markers and transcription factor mRNAs were also examined. The changes in EMT protein markers and transcription factor mRNAs in A549 cells were approximately similar to that in the group treated with Smad2/3 and Src inhibitors. The levels of vimentin, fibronectin and mRNAs decreased by approximately 50%, whereas that of E-cadherin increased by 2-fold compared with those in the cells under hypoxia. The change in the three other groups was found to be smaller than that in the group treated with Smad2/3 and Src inhibitors. The levels of vimentin, fibronectin and mRNAs were reduced by approximately 30%. Hence, EMT is inhibited in A549 cells when the activation of Smad2/3, Src, p38, ERK, and JNK proteins is inhibited under hypoxia (Figure 2B).

**Figure 3:**
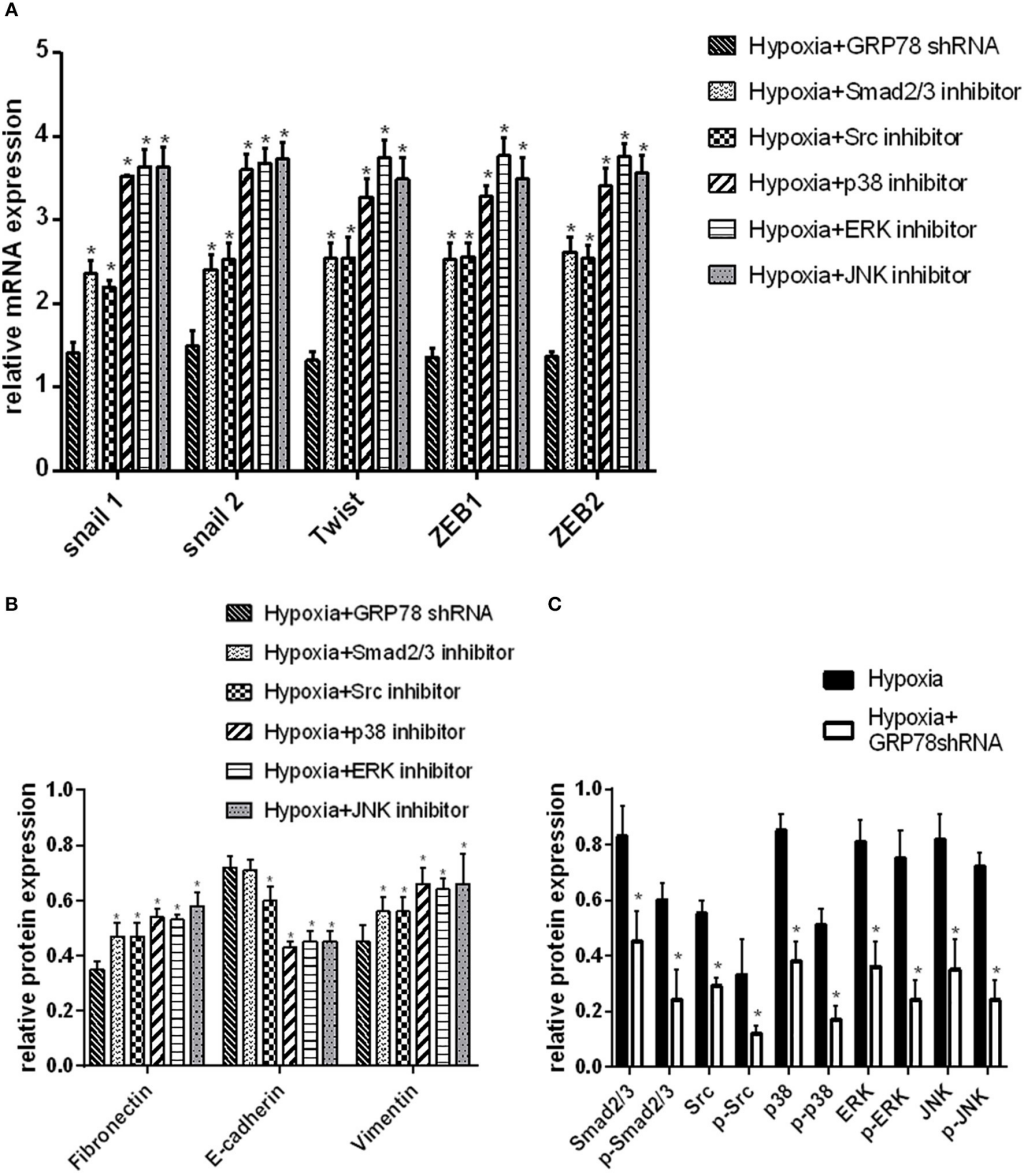
GRP78 is the upper reaches of the Smad2/3, Src and MAPK pathways in hypoxia-induced EMT in A549 cells. **(A)** The mRNA relative value (control group) of EMT transcription factors is plotted (mean ± SD in three separate experiments). *P < 0.05, compared with EMT transcription factors in A549 cells under hypoxia condition separately inhibited by Smad2/3, Src, p38, ERK, and JNK inhibitors, the expression is higher than that in GRP78 knockdown A549 cells under hypoxia. **(B)** The protein relative value (GAPDH) of EMT markers was examined by Western blot analysis and plotted (mean ± SD in three separate experiments). *P < 0.05, compared with EMT markers of A549 cells under hypoxia condition separately inhibited by Smad2/3, Src, p38, ERK, and JNK inhibitors, the expression is higher than that in GRP78 knockdown A549 cells in the hypoxia. **(A,B)** indicate that the inhibition effect of GRP78 silencing is more powerful than those of the five other inhibitors. **(C)** The protein relative values (GAPDH) of Smad2/3, Src, p38, ERK, JNK, and their phosphorylated forms were examined by Western blot analysis and plotted (mean ± SD in three separate experiments). *P < 0.05, compared with A549 cells under hypoxia, the expression levels of Smad2/3, Src, p38, ERK, JNK, and their activation forms decrease compared with those in GRP78 knockdown A549 cells under hypoxia. After GRP78 silencing, the expression and activation of these proteins are inhibited significantly.

**Figure 4:**
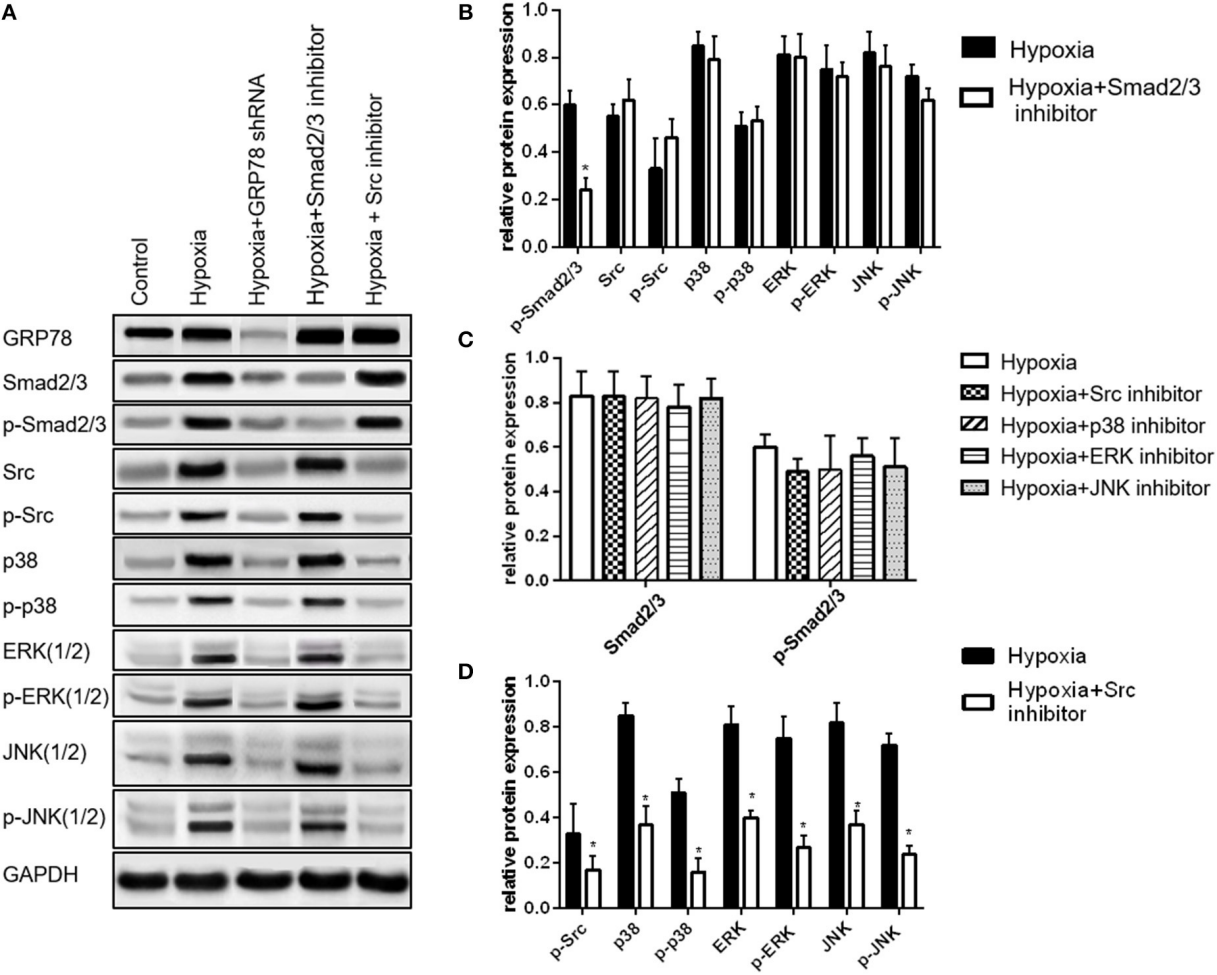
Smad2/3 and Src/MAPK are two dependent signaling pathways in hypoxia-induced EMT in A549 cells. **(A)** Smad2/3, Src, p38, ERK, JNK, and their phosphorylated forms were examined by Western blot analysis. **(B)** Compared with the expression levels of Src, JNK, p38, ERK, and their activation forms in A549 cells under hypoxia, their expression levels do not change after inhibiting the activation of Smad2/3. **(C)** Compared with the expression levels of Smad2/3 and p-Smad2/3 in A549 cells under hypoxia, their expression does not change after inhibiting the activation of Src, JNK, ERK and p38. **(D)** *P < 0.05, compared with the expression levels of p38, ERK, JNK (three forms of MAPK) and their activated forms in A549 cells under hypoxia, their expression levels decrease after inhibiting the activation of Src. MAPK is the downstream pathway of Src.

The changes in the expression of EMT protein markers were compared in A549 cells transfected by GRP78shRNA or treated by different protein inhibitors under hypoxia condition. The changes in the mRNA expression of EMT markers and signaling molecules were the most evident in GRP78 knockdown cells (*P* < 0.05 compared with the other groups, Figures 3A,B). Similar results were obtained on the protein expression of signaling molecules (Smad2/3, Src, p38, ERK, and JNK). After GRP78 silencing, the expression of Smad2/3, Src, p38, ERK, and JNK and their phosphorylated proteins in hypoxia cells was significantly inhibited compared with that in the vehicle control under hypoxia (*P* < 0.05, Figures 3C, 4A).

Different effects were observed on the expression of the signaling molecules after inhibition of a particular pathway. After Smad2/3 inhibition, the expression of the four other signaling molecules did not significantly change (*P* > 0.05, Figures 4A,B). After inhibition of Src, JNK, ERK, and p38 pathways, the expression of Smad2/3 was not significantly changed (P>0.05, Figures 4A,C). After inhibiting Src, the activation of p38, ERK, and JNK (MAPK pathway) was also inhibited (*P* < 0.05, Figures 4A,D).

## Discussion

This study shows that GRP78 highly expressed under hypoxia condition is likely to play an essential role in hypoxia-induced EMT in A549 cells. This main finding is supported by the following observations: (1) the expression of GRP78 was significantly elevated under hypoxia condition and closely associated with the changes in the EMT markers; (2) GRP78 silencing significantly inhibited hypoxia-induced EMT markers; and (3) GRP78 silencing inhibited the expression of several signaling molecules, especially Smad2/3. This work is the first to demonstrate that GRP78 has a causal relationship with hypoxia-induced EMT in lung adenocarcinoma. Hence, targeted inhibition on GRP78 might could hamper EMT, which could further inhibit metastasis and overcome therapeutic resistance.

GRP78 was highly expressed in lung cancer cells under hypoxia condition; this finding is consistent with those reported by Song and Pi (25, 26). Chronic hypoxia induced GRP78 in human cancer cells possibly through the protein kinase C-epsilon/ERK/AP-1 signaling cascade (25).

A causal relationship between high GRP78 expression and EMT was confirmed by the GRP78 knock-down experiment. A very strong correlation was found between changes in the expression of GRP78 and EMT markers. Previous studies suggested that other methods for silencing GRP78 could inhibit EMT. For example, neutralization of endogenous GRP78 on the cell surface with the anti-GRP78 antibody inhibited the ability of adhesion and invasion of hepatocellular carcinoma cell lines Mahlavu and SMMC7721 (19). The mitigation of GRP78 up-regulation by using short hairpin RNA or treatment with the small molecule IT-139 inhibited metastatic growth in the lung microenvironment in four highly metastatic cell line models (three human osteosarcomas and one murine mammary adenocarcinoma) (20). However, no rational interpretation is available regarding the inconsistency on the relationship between GRP78 expression and EMT in colorectal cancer.

The mechanism of GRP78 downstream signaling for EMT promotion has been demonstrated. Cell surface GRP78 can accelerate breast cancer cell proliferation and migration by activating STAT3 (27). We found two key molecular pathways (Smad2/3 and Src/MAPK) of GRP78 that may play an important role in hypoxia-induced EMT by using multiple protein inhibitors. These findings were consistent with those of previous works. Li et al. reported that overexpressing or knocking down GRP78 induced the corresponding activation or inhibition of the Smad2/3 pathway in colon cancer cells (28). Zhao et al. reported that GRP78 interacted directly with Src, thereby promoting the phosphorylation of Src in hepatocellular cancer cells (29). Tanjore et al. also suggested that the combination of the Smad2/3 inhibitor (SB431542) and the Src kinase inhibitor (PP2) blocked the EMT of alveolar epithelial cells induced by ER stress inducer tunicamycin, which also induced high GRP78 expression (30). In the present study, the activation of the Smad2/3 and Src/MAPK pathways follows the same trend with the up-regulation of GRP78; moreover, knockdown of GRP78 inhibited the activation of Smad2/3 and Src, suggesting a causal link between GRP78 and activation of the two pathways in lung cancer. Smad2/3 inhibition did not interact with the inhibition of Src, p38, ERK, and JNK. By contrast, inhibiting the activation of Src was accompanied by the inhibition of p38, ERK, and JNK. Hence, the Smad2/3 and Src/MAPK pathways are two independent downstream signaling pathways of GRP78 during hypoxia-induced EMT in A549 cells. However, we did not perform the knockdown experiment of Smad2/3 and Src, and the co-immunoprecipitation experiment; as such, we cannot provide additional evidence for such link. And the control shRNA was not applied in the present study, which might limiting its evidence.

Other pitfall of the present study is that we have not explored the relationship between GRP78 and HIF1a, which is a key regulator on hypoxia induced EMT. But there are some evidences in other cell lines, that the expression of GRP78 is regulated by HIF1a (31). What's more, all the results in the study are limited to one cell line, limiting its evidence.

**Figure 5:**
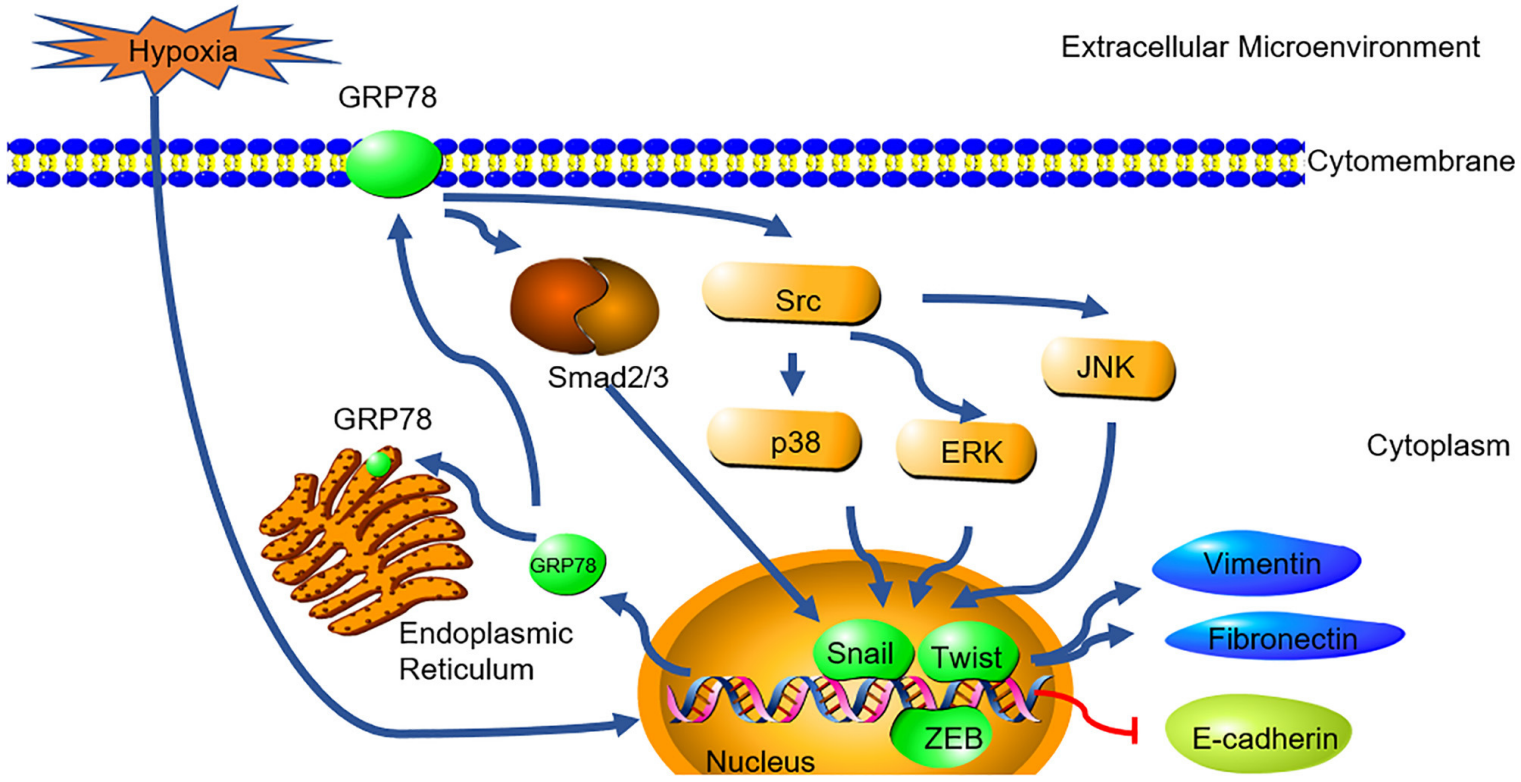
GRP78 mediates hypoxia-induced EMT through smad2/3 and SRC/MAPK signaling. Hypoxia induced the high expression of GRP78. High expressed GRP78 activated the smad2/3 and Src/MAPK pathway. The pathway activated the expression of EMT transcription factor (snail, Twist and ZEB) and collaborated with them to induce the expression of Fibronectin, Vimentin and inhibit the expression of E-cadherin.

In summary, this study demonstrated the possible causal link between GRP78 and hypoxia-induced EMT in A549 cells (Figure 5). Together with its roles in anti-apoptosis and chemoresistance, it indicates that GRP78 might be a potential target for treatment of lung adenocarcinoma. Further, studies are needed to elucidate the exact mechanisms involved in the GRP78-EMT pathway in hypoxia and their relevant clinical significance.

## Data Availability

All datasets generated for this study are included in the manuscript and/ or the supplementary files.

## Author Contributions

L-ZL and L-LS: designed the experiments. L-LS, C-MC, and JW: performed the experiments. JZ and C-ZY: analyzed the data. L-LS and C-ZY: prepared the figures. L-LS and JZ: wrote the main manuscript. All authors reviewed the manuscript.

### Conflict of Interest Statement

The authors declare that the research was conducted in the absence of any commercial or financial relationships that could be construed as a potential conflict of interest.
