# Addendum: Glucose-Regulated Protein 78 Signaling Regulates Hypoxia-Induced Epithelial–Mesenchymal Transition in A549 Cells

**DOI:** 10.3389/fonc.2021.637227

**Published:** 2021-06-01

**Authors:** Ling-Ling Sun, Chang-Ming Chen, Jue Zhang, Jing Wang, Cai-Zhi Yang, Li-Zhu Lin

**Affiliations:** Integrative Cancer Centre, The First Affiliated Hospital of Guangzhou University of Chinese Medicine, Guangzhou, China

**Keywords:** lung cancer, lung adenocarcinoma, epithelial mesenchymal transition, hypoxia, glucose-regulated protein 78, GRP78

We thank the reviewers for their queries regarding the suitability of GAPDH, a protein that increases in expression during hypoxia, as a control in our hypoxia experiment.

We have included additional commentary on this issue in the discussion section.

As for typical controls used in hypoxia experiments that induce the epithelial–mesenchymal transition (EMT), we found that most articles used beta-actin (1–3) and GAPDH (4–7) as reference proteins, while a few articles used tubulin (8). However, it seems that there are no absolute ideal controls specifically when it comes to analyzing the cellular changes like cell death, proliferation, mutagenesis and transition. GAPDH is a rate limiting enzyme in glycolysis. External stimuli such as hypoxia can enhance the expression of GAPDH in some cells (9, 10). However, the expression of GAPDH might be unchanged in cancer cells such as human glioblastoma under hypoxic conditions (11), despite the metabolic phenotype of cancer cells being accelerated glycolysis, even under normoxic conditions (12). On the other hand, beta-actin and beta-tubulin are closely related to EMT and they are also not ideal controls in this circumstance. Beta-actin plays a key role in cell migration, which is always accompanied by the EMT process (13). The expression and function of beta-actin is expected to change during the EMT process (14). Beta-tubulin is partially under the negative control of miR-200, a family of micro-RNAs playing a major role in EMT (15). And coordinated regulation exists between β-tubulin and EMT protein ZEB1 (16). In addition to hypoxia, many other stimuli or interventions can affect the metabolic phenotype and EMT process of cancer cells (17–19). Thus, in such a scenario, it’s required to have a reasonable control which is further checked as internal control within the study in different conditions of the study, then that can be utilized to draw conclusions of test proteins level change. We also asked ourselves whether the use of GAPDH has led to a false positive conclusion in the present study. We argue that a false positive is unlikely for the following reasons. Firstly, hypoxia stimulates the expression of GAPDH. If we adjusted the expression of GAPDH, then the expression of GRP78 should increase. Secondly, whether using beta-actin (20, 21) or beta-tubulin (22) as control, GRP78 was also highly expressed in hypoxic cells. Thirdly, the signaling of smad2/3 and SRC will not be influenced by the use of GAPDH, as the control was also exposed to a hypoxic environment. As a result, we do not think that the conclusion of the present study is a false positive result due to the application of GAPDH.
